# The Impact of Free Sugar on Human Health—A Narrative Review

**DOI:** 10.3390/nu15040889

**Published:** 2023-02-10

**Authors:** Kerri M. Gillespie, Eva Kemps, Melanie J. White, Selena E. Bartlett

**Affiliations:** 1 School of Clinical Sciences, Faculty of Health, Queensland University of Technology, Kelvin Grove, QLD 4059, Australia; 2 College of Education, Psychology and Social Work, Flinders University, Bedford Park, SA 5042, Australia; 3 School of Psychology and Counselling, Faculty of Health, Queensland University of Technology, Kelvin Grove, QLD 4059, Australia

**Keywords:** sugar, high-fructose corn syrup, fructose, sugar-sweetened beverage, cognition, obesity, coronary heart disease, diabetes, microbiome, neuroinflammation

## Abstract

The importance of nutrition in human health has been understood for over a century. However, debate is ongoing regarding the role of added and free sugars in physiological and neurological health. In this narrative review, we have addressed several key issues around this debate and the major health conditions previously associated with sugar. We aim to determine the current evidence regarding the role of free sugars in human health, specifically obesity, diabetes, cardiovascular diseases, cognition, and mood. We also present some predominant theories on mechanisms of action. The findings suggest a negative effect of excessive added sugar consumption on human health and wellbeing. Specific class and source of carbohydrate appears to greatly influence the impact of these macronutrients on health. Further research into individual effects of carbohydrate forms in diverse populations is needed to understand the complex relationship between sugar and health.

## 1. Introduction

Noncommunicable diseases (NCDs) are chronic and largely preventable conditions, such as diabetes, heart disease, kidney disease, cancer, and mental health disorders [1]. NCDs account for around 74% of deaths globally and place an enormous financial burden on healthcare services and households [1,2]. Lifestyle factors such as weight, diet, physical activity, and substance use are major contributing factors to the burden of preventable disease. Obesity is an increasing global health concern, occurring in 13% of the world’s population, and is considered a major risk factor for NCDs, mortality, and reduced quality of life [3]. Chronic conditions are frequently associated with multi-comorbidity and polypharmacy, increasing the risk of a ‘prescribing cascade’, drug side-effects, and drug–drug interactions [4,5]. Lifestyle modification for treatment and prevention of these diseases is optimal. A great deal of evidence supports lifestyle changes, such as improved diet for prevention of obesity, diabetes, heart disease, and cognitive decline [6]. However, the role of specific diets and macronutrients is still disputed.

Studies investigating the impact of sugar consumption on human health have been ongoing since the mid-20th century. However, there is continued debate regarding the role sugar plays in physical, neurological, and cognitive health. Excessive sugar consumption has been implicated in obesity, metabolic disorders, diabetes, cardiovascular disease, cancer, depression, and cognitive impairment [7,8,9,10]. Several researchers have criticised these claims as exaggerated or misleading, claiming that sugar is no more detrimental than any other source of dietary energy, and even promoting its benefits for health and cognitive function [11,12,13]. The aim of this narrative review is to collate and summarise the existing research related to impacts of added dietary sugars on human health cognition and mood and to provide an overview of the findings (see Table 1 for a summary of findings). The paper will outline evidence regarding the role of added sugars in several chronic conditions and highlight gaps in the current knowledge.

## 2. Background

### 2.1. Controversies

Since the discovery of the role of diet in diseases such as scurvy and rickets in the early 1900s and isolation of the first vitamin (Thiamine) in 1926, nutrition has become an exponentially expanding industry and field of research [64]. The 1950s saw growing evidence of a dietary role in the increasing rates of coronary heart disease (CHD) witnessed at the time [65,66]. This led to two divergent hypotheses of CHD that have also been presented as explanations for obesity, diabetes, and non-alcoholic fatty liver disease [67,68,69]. These contrary propositions are: (1) the added sugar hypothesis proposed by John Yudkin; and (2) the saturated fat and cholesterol theory proposed predominantly by Ancel Keys, Frederick Stare, and Mark Hegsted [66,70]. By the 1980s, the sugar hypothesis had been eclipsed by the more popular fat theory, and a wave of low-fat guidelines and food alternatives swept the globe for the following four decades [71].

The past ten years have observed an increase in articles suggesting that evidence linking sugar to coronary disease was downplayed or suppressed due to pressure from the Sugar Research Foundation (SRF) (now the Sugar Association) [66,72]. The claims cite several studies funded by the SRF that downplayed the role of sugar and highlighted the role of fats in CHD [73]. While these claims have been contested by some, a lack of transparency around funding and research at the time makes them difficult to confirm or contest. Aside from these controversies, both the ‘added sugars’ and ‘dietary fats’ hypotheses of illness have suffered criticism for their reductionist approaches to nutritional causes of disease, i.e., being primarily affected by a single macronutrient.

The Framingham Heart Study (a cohort study of over 14,000 people from three generations that started in 1948) brought attention of sugar as a major factor back into the public eye. This study found that frequent consumers of sugar-sweetened beverages (SSBs) had significantly increased liver fat and dysbiosis (decreased high-density lipoprotein (HDL) and increased triglyceride and cholesterol levels) [40,74].

### 2.2. Trends in Sugar Consumption

The association of fat with cardiovascular disease and obesity in the mid- to late 20th century led to a reduction in fat consumption and an increase in carbohydrates and refined sugars [74]. High-fructose corn syrup accounted for less than 1% of caloric sweeteners in the 1970s but increased to 42% by 2000 [75]. Estimated total sugar intake between 1977 to 1998 increased from 235 to 318 kcal per day [75]. According to food availability data, added sugars and sweeteners reached a peak of over 69 kg (153 pounds) per person per year in the USA in 1999 [76]. These changes in diet coincided with a dramatic rise in obesity, diabetes, and cardiovascular disease [77].

Added sugars refers to sugars that are added in food preparation or manufacturing, such as glucose, fructose, sucrose (a sugar molecule made from glucose and fructose combined), and hydrogenated starch hydrolysates (high-fructose corn syrup) [78]. The World Health Organization (WHO) and the Scientific Advisory Committee on Nutrition (SACN) use the term ‘free’ sugars, which also includes all sugars that are naturally present in honey, fruit juices, and syrups. This is generally not considered to include sugars found within the cellular structure of foods, such as dairy foods, or the carbohydrates found in nuts, fruit, cereal grains, or vegetables.

The turn of the century witnessed a modest decline in added sugar intake. A report by the US Department of Agriculture noted a reduction in added sugars and sweeteners by 14% between 1999 and 2014 [76]. Trends from the US 2001 to 2018 National Health and Nutrition Examination Survey (NHANES) highlighted this reduction, albeit observed only in younger adults (aged 19–50 years) from a mean of 96.6 g to 72.3 g per day, including a reduction in SSBs from 49.7% of daily sugar intake to 37.7% [79]. A similar reduction in sugar was observed in Australia and New Zealand between 1995 and 2011, with the proportion of dietary energy from free sugars declining from 12.5% to 10.9% [80]. The greatest contributor to this decline was again observed in children and young adults. These declines in sugars were accompanied by only a minimal 1% increase in dietary fats [81]. Despite the decline, global sugar consumption is still high and well above the recommended 5% or 10% of daily energy intake [82,83]. SSBs are still the main source of daily added sugars in most Western countries [84].

Growing evidence linking free or added sugars to obesity, heart disease, and dental caries prompted introduction of sugar guidelines by the WHO, the American Heart Association (AHA), and the UK National Health Service (NHS), among others. A 2014 systematic review highlighting the significant impact of sugar intake on dental caries [85] was instrumental in formation of the WHO guidelines for recommended daily sugar intake. The AHA and WHO recommend no more than 10% of total calories be added sugars; that is approximately 200 calories, 50 g, or 12 teaspoons for an average adult [28]. However, both these organisations note that a limit of 5% of total calories per day would improve health outcomes. The NHS guidelines recommend that free sugars not make up more than 5% of calories from food or exceed 30 g per day [82].

### 2.3. Obesity

Rates of global obesity have tripled since 1975, with the WHO estimating that 13% of adults were obese and 18% of children were overweight or obese in 2016 [3]. Australian estimates of obesity are even higher, at 31% of adults and 8.2% of children and adolescents in 2017–18 [86]. Obesity significantly increases risk of several non-communicable diseases, such as diabetes, cancer, cardiovascular disease, dementia, obstructive sleep apnoea, stroke, osteoarthritis, and liver disease [87]. Obesity research has suffered from the same fat or sugar conundrum as CHD. The ongoing debate remains primarily focused on whether the cause of overweight and obesity is excess sugar, excess fat, or an excess total calorie intake (the “Energy Balance Model”) [88,89].

The declines in sugar consumption in recent years, alongside the continued increase in obesity, may suggest that sugar is not the major contributor to weight gain. Bentley [90] suggests that this shift is caused by a generational delay. As childhood consumption tends to predict adult obesity, the increases in adult obesity (i.e., ages 40 to 70 years, for example) reflect poor diets of children in the 1950s through to the 1980s. This reasoning is further supported by reductions in sugar being observed predominantly in children and younger adults. If Bentley’s argument is correct, we should see levels of obesity begin to decline as the children who have grown up with reduced sugar in their diets grow into adulthood. Another proposed explanation is the shift from traditional sugars to sugar alternatives (low-calorie, artificial sweeteners), which saw an annual global growth of approximately 5.1% per year between 2008 and 2015 [91]. Recently, studies have begun to investigate the impacts of these sugar alternatives and have found links between specific sweeteners and obesity [92], cardiovascular diseases [93], and cancer [94]. This is a relatively new but growing field of research. Another factor impacting this relationship is the increasing prevalence of inactivity. Global rates of insufficient activity range from 16.3% to 39.1%, with rates increasing in higher-income Western countries [95]. Global, population-based surveys have found that over 80% of adolescents 11–17 years of age were insufficiently physically active in 2016 [96].

Research investigating differences between low-carbohydrate- and low-fat diets has demonstrated varied results [97,98,99,100,101,102,103]. Most of these studies found little or no difference in weight loss when comparing isocaloric reductions in fat to carbohydrates. The main differences between diets were a more profound improvement in high-density lipoproteins (HDL) and lipid profiles in low-carbohydrate diets and a greater reduction in low-density lipoproteins (LDL) in low-fat diets [99,100,104]. These findings indicate the potential for individualised dietary therapies to preferentially target different lipid and glycaemic treatment goals depending on diagnosis or cardiovascular risk profile.

Studies that found improved weight loss after reduced fat intake often had the limitation of failing to classify the type of carbohydrates consumed [102,105]. High-glycaemic-index (GI) carbohydrates (which include white bread, potatoes, sugar, and white rice) are understood to promote postprandial carbohydrate oxidation at the expense of fatty acid oxidation, which has led to increased lipogenesis, insulin resistance, and weight gain in animal models [106]. Conversely, consumption of high-fibre, low-GI carbohydrates has led to reductions in postprandial glucose response, cholesterol, and adiposity [107,108].

The results of large observational studies have been mixed. For example, a large epidemiological study in the UK (N = 132,479) found that obese adults consumed a larger amount of each food group than underweight or normal weight individuals [109]. While sugar consumption was higher in the obese group, a higher proportion of daily kilojoules were consumed as fat (34.3%) than as sugar (22.0%) compared to normal weight participants (33.4% and 24.2%, respectively). The study also found a negative correlation between sugar consumption and adiposity when controlling for age, sex, ethnicity, physical activity, and total energy intake. Another large study of 1165 children and adolescents in the Hellenic National Nutritional Health Survey found that children and adolescents consuming ≥ 10% energy intake from added sugars were 2.57 times more likely to be overweight or obese than those consuming less than 10% [110]. Discrepancies may be due to methodological differences, or at least partially due to limitations of self-report measures, which are vulnerable to recall bias and other confounders. Food frequency questionnaires in particular have been found to have variable reliability [111]. Ravelli and Schoeller [112] found that not only do participants tend to underreport energy intake but this underreporting tends to be greater as BMI increases.

The evidence for an impact of sugars on obesity appears to be stronger when investigating the impacts of SSBs (as opposed to total sugar intake or other forms of carbohydrate). Numerous studies have been conducted, with multiple systematic reviews and meta-analyses concluding that SSB consumption promotes weight gain [113,114,115,116]. Sucrose and high-fructose corn syrup from SSBs are the major source of fructose in our diets, which are thought to have a more detrimental impact on physical and neurological health given the unique way they are metabolised in the body. Fructose metabolism is similar to glycolysis except that it bypasses the regulatory step of phosphofructokinase, which is able to regulate glycolysis through allosteric inhibition [117]. Unlike glucose, fructose is insulin-independent and metabolized predominantly in the liver. It has also been implicated in increased hepatic de novo lipogenesis and hypertriglyceridemia, important characteristics of metabolic syndrome and non-alcoholic fatty liver disease.

Many studies to date have found a strong link between sugar consumption and obesity. However, meta-analyses have revealed that increased sugar consumption is associated with increased energy intake in general, giving more credence to the notion that higher energy intake accounted for by all calories, rather than just sugar, is the cause of the obesity epidemic. International data show that carbohydrate consumption is declining but diet quality is still poor and calorie intake high [118]. Rather than just a case of too much sugar, could it be a case of too much food intake overall? Further research into the differential mechanisms of fructose and the unique roles of different fat and carbohydrate classifications may reveal improved dietary targets for obesity management.

### 2.4. Diabetes

Diabetes is the disease most commonly associated with sugar consumption. Whether sugar is a unique cause or contributor to diabetes is another contested issue. Many prospective and retrospective studies have been conducted with varying and inconsistent results [119,120]. However, the majority of research conducted has found a positive association between sugars [121], particularly fructose and SSB consumption [35,119,122], and risk for diabetes type 2 (T2DM) (see Table 2). Several animal and human studies have observed impaired insulin signalling and increased fasting glucose and insulin resulting from SSB consumption [61,123,124]. The association has been particularly evident in women but not always observed in male cohorts [125,126,127].

Numerous systematic reviews and meta-analyses have found strong associations between SSB consumption and T2DM incidence [37,128,129,130,131,132]. An econometric model of repeated cross-sectional data from 175 countries indicated that sugar was significantly correlated with diabetes risk in a dose-dependent manner, with reductions in sugar associated with a decline in diabetes incidence [133].

While many studies have found significant results, some have found no, or inconsistent, relationships [120]. It should be noted, however, that studies finding no relationship between added sugars and diabetes risk tended to be of a shorter duration: four weeks [20] to six years follow-up [134,135]. In contrast, studies that supported the impact of fructose, glucose, and SSB intake on increased diabetes risk tended to be of longer follow-up duration, at least 10 years or more [80]. A four-week study by Lê et al. [20] that found no insulin resistance did identify increases in fasting TG, LDL, and leptin, which are all implicated in development of diabetes and cardiovascular disease. This suggests that added sugars may have numerous systemic effects that can be observed early, prior to development of chronic diseases such as T2DM.

The effect of time is evidenced by the findings of the Nurse’s Health Study, where an 8-year follow-up by Colditz et al. [135] showed no relationship between sucrose and diabetes. However, a subsequent 18-year follow-up of the same cohort showed increased risk of T2DM [136]. Nevertheless, one 12-year follow-up study found no relationship between high-glycaemic-index (GI) carbohydrates and diabetes risk [137].

Studies that found no relationship between added sugars and subsequent diabetes risk were predominantly investigating glucose or sucrose intake [21,63,135,138]. The majority of studies that identified a significant positive relationship between added sugar and diabetes diagnosis or risk factors measured intake of fructose or fructose-containing beverages [21,29,31,32,63], which may indicate a unique deleterious effect of fructose over other forms of carbohydrates. For example, a study in overweight and obese human subjects found that ten weeks of fructose consumption, but not glucose consumption, decreased insulin sensitivity [21]. This issue is further complicated by the fact that natural fructose (from fruit) may be associated with a reduced risk of T2DM, while fructose from SSBs (high-fructose corn syrup) is associated with an increased risk [126,139].

A commonly found protective factor for diabetes risk was consumption of fruit or green leafy vegetables and high-fibre, low-GI foods [136,140,141,142]. However, protective factors, such as fibre and exercise, were not always assessed and accounted for in analyses, and this may account for some of the inconsistencies in results. Some studies investigating the relationship between fat and T2DM found some protection from diabetes attributable to certain fats, particularly dairy [143,144]. Considering obesity is a known risk factor for diabetes and high-fat diets may lead to obesity and other health issues, it is not suggested that long-term high-fat diets would be beneficial in this cohort. Further studies that measure comprehensive macronutrient intake and exercise over longer time periods of at least ten years may provide clarification. While data weigh in favour of sugar being a risk factor for diabetes, more knowledge regarding sex-specific relationships and the independent effect of sugar type is required to further our understanding of this relationship.

**Table 2:** Findings from published reports on the effects of free or added sugars on chronic disease in human subjects.

Authors and Year	Design	Timeframe/ Follow-Up	Subjects	Measures	Intervention/ Independent Variable	Findings
Ahmadi-Abhari et al., 2014. [138]	Case-control study.	6.3y (mean)	Aged 40–79y; n = 749 diabetes cases; n = 3496 controls.	FFQ (total sugars, fructose, glucose, lactose, sucrose, maltose). Physical assessment	↑ Fructose and glucose	↓ Risk of T2DM
Bazzano et al., 2008 [136].	Prospective cohort study	18y	Female registered nurses (NHS); aged 30–55y; n = 71,346	FFQ (fruit juice, whole fruit, whole vegetables) Self-reported T2DM	↑ Fruit juice	↑ T2DM risk (Whole fruits and green leafy vegetables decreased T2DM risk)
Bernstein et al., 2012 [145].	Prospective cohort study	NHS: 28y HPFS: 22y	NHS: Women aged 30–55y; n = 84,085 HPFS: men aged 40–75y; n = 43,371	FFQ (SSB = soft drinks, fruit juice) Self-reported T2DM	↑ SSB	↑ Stroke risk
Colditz et al., 1992 [135].	Prospective cohort study	6y	Female registered nurses (NHS); aged 30–55y; n = 84,360	FFQ (sucrose) Self-reported T2DM	Sucrose	-Sucrose was not related to T2DM risk.
De Koning et al., 2012 [15].	Prospective cohort study	22y	Adult males; n = 42,883	FFQ (SSB = SD, fruit punch, fruit drinks) Self-reported CHD Biomarkers (n = 18,225)	↑ SSB	↑ CHD risk ↑ TG ↑ Inflammatory markers ↓ HDL ↓ Leptin
Dhingra et al., 2007 [41].	Prospective cohort study	4y (mean)	Adults (FHS); n = 6039	FFQ (SD)	↑ SD	↑ MetS prevalence
Drouin-Chartier et al., 2019 [29].	Prospective cohort study	NHS: 1986–2012 NHS II: 1991–2013 HPFS: 1986–2012	NHS: women aged 30–55y; n = 76,531 NHS II: women aged 25–42y; n = 81,597 HPFS: men aged 40–75y; n = 34,224	FFQ (SSB = soft drinks, fruit juice) Self-reported T2DM	↑ SSB	↑ T2DM risk
Eshak et al., 2012 [25].	Prospective cohort study	18y	Aged 40–59; n = 43,149)	FFQ (SD) Medical records	↑ SD	↑ Total stroke risk ↑ Ischaemic stroke risk in women ↓ Ischaemic stroke risk in men
Eshak et al., 2013 [126].	Prospective cohort study	10y	Aged 40–59y; n = 27,585	FFQ (soft drink, 100% fruit juice, vegetable juice) Self-reported T2DM	↑ SD	↑ T2DM risk in women -No relationship between fruit/vegetable juice and T2DM
Fagherazzi et al., 2013 [30].	Prospective cohort study	14y	Adult women; n = 66,118	FFQ (SSB) Self-reported T2DM	↑ SSB	↑ T2DM risk
Ferreira-Pego et al., 2016 [42].	Prospective cohort study	3.24y (median)	Adults; n = 1868	FFQ (SSB) Physical assessment	>5 servings SSB per week	↑ MetS risk
Fung et al., 2009 [16].	Prospective cohort study	24y	Adult females (NHS); n = 88,520	FFQ (SSB) Medical records	↑ SSB	↑ CHD Incidence
Haslam et al., 2020 [19].	Prospective cohort study	12.5y (mean)	FHS Offspring: n = 3146 FHS Third generation: n = 3584	FFQ (SSB) Physical assessment	↑ SSB	↓ HDL ↑ TG
Hirahatake et al., 2019 [31].	Prospective cohort study	30y	Aged 18–30; n = 4719	FFQ (SSB) Interviews Pathology results	↑ SSB	↑ T2DM risk
Huang et al., 2017 [32].	Prospective cohort study	8.4y (mean)	Adult females (aged 50–79 years; n = 64,850)	FFQ (SSB) Self-reported T2DM	↑ SSB	↑ T2DM risk
Romaguera et al., 2013 [33]	Retrospective Case-Cohort study.	NA	Adults; n = 12,403 diabetes cases; n = 16,154 controls	FFQ (SD, juice, nectar)	↑ SSB	↑ T2DM risk
Janket et al., 2003 [134].	Prospective cohort study.	6y (mean)	Aged 45 years and over; n = 38,480	FFQ (sucrose, fructose, glucose, lactose)	Total sugar intake	No relationship between sugars and T2DM incidence.
Janzi et al., 2020 [26].	Prospective cohort study.	19.5y (mean)	Adults; n = 16,781	FFQ (Added sugar, SSB, sugary treats). Physical assessment Interview	↑ SSB	↑ Stroke risk
					↑ Added sugar	↑ Stroke risk ↓ Aortic stenosis
					↑ Sugary treats	↓ Coronary events
Jebril et al., 2020 [121].	Cross-sectional survey	NA	Adults; n = 1000	FFQ (added sugar) Physical health assessment Medical records	Added sugar intake	-No relationship between sugar and diagnosed T2DM. -Positive relationship between sugar and undiagnosed T2DM.
Larsson et al., 2014 [27].	Prospective cohort study	10.3y (mean)	Aged 44–83y; n = 68,459	FFQ (SSB) Medical records/death register	≥2 servings SSB per day	↑ Total stroke ↑ Cerebral infarction
Le et al., 2006 [20].	Repeated measures experimental study.	4 weeks	Adult males; (n = 7)	High-fructose diet (1.5 g/kg).	Fructose (1.5 g/kg)	↑ LDL ↑ TG ↑ Leptin ↓ Non-esterified fatty acids -No change in insulin resistance
Lowndes et al., 2015 [63].	Randomised Parallel group study	10 weeks	Aged 20–60y; BMI = 21–35 kg/m^2^; n = 198 (28–34 per study group)	Consumption of milk containing HFCS, fructose, glucose, and sucrose, contributing 18%, 9%, 9%, and 18% of energy intake compared to controls.	Fructose 9%	↑ Insulin ↑ Hepatic insulin resistance ↑ Weight (for all sugar intervention groups)
Maersk et al., 2012 [39].	Randomised Parallel group study	6 months	Overweight adults; aged 26–40 years; n = 47 (SD group, n = 10)	Dietary record Physical assessment	1 Litre SD per day (50% glucose, 50% fructose)	↑ Visceral adipose tissue ↑ Liver fat ↑ Skeletal muscle fat ↑ TG ↑ Total cholesterol
Miao et al., 2021 [28]	Prospective cohort study	16y (mean)	Adults (FHS); n = 1384	FFQ (SSB) Hospital admission records	↑ SSB	↑ Stroke risk
Montonen et al., 2007 [146].	Prospective cohort study	12y	Ages 40–60y; n = 4304	FFQ (total sugars, fructose, glucose, lactose, sucrose, maltose).	↑ Fructose and glucose	↑ T2DM incidence
O’Connor et al., 2015 [34].	Prospective cohort study	10.8y	Aged 40–79y; n = 25,639	7-day food diaries (SD, fruit juice, sweetened tea/coffee, sweetened milk) Self-reported T2DM Medical records	↑ SD or sweetened milk drinks	↑ T2DM risk (No effect of fruit juice or tea/coffee)
Odegaard et al., 2010 [35].	Prospective cohort study	5.7y (mean)	Aged 45–74; n = 43,580	FFQ (SSB = Soft drink or fruit/vegetable juice).	↑ SSB	↑ T2DM risk
Pacheco et al., 2020 [17].	Prospective cohort study	20y	Adult women; mean age 52.1y; n = 106,178	FFQ (SSB = caloric soft drinks, sweetened water, fruit drinks) Medical records	↑ SSB	↑ CVD risk
Palmer et al., 2008 [36].	Prospective cohort study	10y	Adult women; n = 43,960	FFQ (SSB = soft drink and juice) Self-report T2DM	↑ SSB	↑ T2DM risk (Orange and grapefruit juice not associated with T2DM risk)
Papier et al., 2017 [125].	Prospective cohort study	8y	Adults (n = 39,175)	FFQ (SSB) Self-report T2DM	↑ SSB	↑ T2DM risk in women
Park et al., 2022 [40].	Prospective cohort study	FHS Offspring: 6y FHS Third generation: 6.2y	FHS Offspring: Adults; mean age 62.8y; n = 691 FHS Third generation: Adults; mean age 48.4 years; n = 945.	FFQ (SSB) Physical assessment	↑ SSB	↑ NAFLD incidence ↑ Liver fat
Paynter et al., 2006 [147].	Prospective cohort study	9y	Middle-aged adults; n = 12,204	FFQ (SSB = fruit punch, non-diet soft drink, orange juice, grapefruit juice)	SSB	-No relationship between SSB and diabetes risk (with or without juice)
Rahman et al., 2015 [14].	Prospective cohort study	11.7y (mean)	Men aged 45–79; n = 42,400	FFQ (SSB) Medical records/death register	↑ SSB	↑ HF risk
Sakurai et al., 2014 [127].	Prospective cohort study.	7y	Men aged 35–55y; n = 2037	FFQ (SSB) Pathology results	↑ SSB	-No effect on T2DM risk
Schulze et al., 2004 [37].	Prospective cohort study.	8y	Adult women; n = 91,249	FFQ (SSB)	↑ SSB	↑ T2DM risk ↑ Weight
Shin et al., 2018 [43].	Cross sectional	NA	Adults; n = 12,112	FFQ (SSB = soft drinks, fruit juices, sweetened rice drinks). Physical assessment	↑ SSB	↑ MetS risk in women ↑ Obesity prevalence
Stanhope et al., 2009 [21].	Double-blinded parallel arm study with matched subjects.	10 weeks	Aged 40–72y; BMI = 25–35 kg/m^2^; n = 32	Consumption of glucose- or fructose-sweetened beverages providing 25% of energy.	Fructose	↑ Increase body fat and weight ↑ Postprandial de novo lipogenesis ↑ Fasting glucose ↑ Fasting insulin ↓ Insulin sensitivity index
					Glucose	↑ Increase body fat and weight ↑ TG ↓ Fasting glucose
Stern et al., 2019 [38].	Prospective cohort study	2.16y (median)	Women aged ≥ 25 years; n = 72,667	FFQ (SD) Self-reported T2DM	↑ SD	↑ T2DM incidence
Welsh et al., 2011 [22].	Prospective cohort study (NHANES subgroup, 1999–2004)	NA	Aged 12 to 18y; n = 2157	FFQ (added sugars) Pathology results	↑ Added sugars	↓ HDL ↑ TG ↑ Fasting insulin in overweight individuals only ↑ Insulin resistance in overweight individuals only
Yang et al., 2014 [18].	Prospective cohort study (NHANES: T1, 1988–1994; T2, 1999–2004; T3, 2005–2010)	14.6y (median)	Adults; n = 11,733 (T1), 8786 (T2), 10,628 (T3); BMI ≥ 18.5 kg/m^2^	FFQ (added sugars) Death register	↑ Added sugars	↑ CVD mortality risk
Yu et al., 2018 [23].	Cross-sectional survey (NHS)	NA	Women aged 30–55 years; n = 8492	FFQ (SSB) Biospecimens	↑ SSB	↑ TG ↓ HDL ↑ Inflammatory biomarkers ↑ Insulin ↓ Adiponectin

Abbreviations: y, years; FFQ, food frequency questionnaire; ↑ increased/increasing; ↓ decreased/decreasing; T2DM, type 2 diabetes mellitus; NHS, Nurse’s Health Study; HPFS, Health Professional’s Follow-up Study; FHS, Framingham Heart Study; SSB, sugar-sweetened beverage; SD, soft drink; CHD, coronary heart disease; TG, triglycerides; HDL, high-density lipoprotein; MetS, metabolic syndrome; LDL, low-density lipoprotein; BMI, Body Mass Index; CVD, cardiovascular disease; NHANES, National Health and Nutrition Examination Survey; HFCS, high-fructose corn syrup.

### 2.5. Heart Disease

Dietary impacts on heart disease have been widely contested over the past 70 years, yet there are still few studies that directly investigate impact of refined carbohydrates on CHD compared to those investigating fats. While low-fat foods dominated our understanding of a healthy diet, few studies have supported that hypothesis. Studies that investigated the impacts of carbohydrates are mixed but more strongly support a refined carbohydrate and SSB contribution to heart disease [18,131] (see Table 2). A meta-analysis of 39 trials found that high sugar intake was significantly associated with increased dyslipidaemia [148]. A large repeated measures longitudinal study across 24 years found that carbohydrates from refined starches and added sugars, as well as trans fats, were significantly associated with higher CHD risk [149]. Conversely, it found that carbohydrates from whole grains and intake of polyunsaturated fatty acids were related to reduced risk of CHD. A similar 22-year study of only men found an increased risk of CHD, dyslipidaemia, and raised inflammatory markers subsequent to SSB consumption [15].

Not all studies have found this same increased risk, however. For example, a large international epidemiological study (N = 135,335) with a shorter median follow-up of 7.4 years found that carbohydrates were related to increased risk of death but not to coronary disease or coronary mortality [150]. However, this study did not classify type of carbohydrates measured, nor was it able to measure trans fats, which may explain the discrepancies with the previous study. Longer time frames may also be required to identify long-term cardiovascular risk. A Swedish population study (N = 25,877) with a mean follow-up of 19.5 years [26] found that added sugar (which specifically excluded sugar from fruits, vegetables, and fruit juice) was positively correlated with stroke and coronary events but negatively correlated with atrial fibrillation and aortic stenosis. This supports the need to further investigate individual macronutrient types (specifically carbohydrate classifications) for their independent impacts on cardiovascular health.

It is well documented that coronary heart disease and cardiovascular diseases are related to hyperglycaemia and insulin resistance. Dyslipidaemia (particularly hypertriglyceridemia) and impaired insulin signalling are strongly associated with heart disease [151,152,153,154]. These conditions have commonly been found following long-term SSB consumption in large-scale human trials [19,22,23,155]. Sprague Dawley rat trials found that fructose, but not glucose, resulted in hyperinsulinemia and hypertriglyceridemia [61,123], indicating a significant factor of carbohydrate source. The findings above indicate that sugars likely play a deleterious role in cardiovascular health, but specific types of carbohydrates and fats can have vastly dissimilar results in terms of mechanism and disease outcome and should, therefore, be considered in future studies.

### 2.6. Cognition

Monosaccharide glucose is the primary energy source for the mammalian brain [156]. The brain requires about 20% of glucose-derived energy provided by basal metabolism [157]. Consistent and tightly regulated glucose metabolism is required for neuronal function, ATP generation, cellular maintenance, and synthesis of neurotransmitters [156,157]. However, it has been proposed that excessive sugar consumption may lead to cognitive impairment and an increased risk of dementia. A growing body of research has observed long lasting impacts of chronic excessive sugar intake on memory, mood, object recognition, and concentration [8,62,158] (see Table 3).

Prior studies have associated increased adiposity (rather than dietary constituents) with reduced cognitive function [159,160], but sugar-induced impairments in cognition have been observed independently to, or prior to, weight gain [7]. Animal studies have identified several similar functional and structural neurological impairments (particularly hippocampal impairment and neuroinflammation) subsequent to high levels of sugar consumption over a four-to-six-week period in adolescents and adults [49,50]. Beecher et al. [10] found that chronic overconsumption of sucrose in adolescent mice over a 12-week period impaired adult episodic and spatial memory and reduced hippocampal cell proliferation. Studies in rats found that early-life high-fructose corn syrup exposure demonstrated long-lasting cognitive and affective alterations in adulthood, along with significant protein abnormalities in the nucleus accumbens (NAcc) [53,62].

Maternal exposure to diets high in sucrose [161] and fructose [162] resulted in spatial cognition deficits and hippocampal alterations in rat offspring. These findings have been replicated in human studies where maternal (pregnant or breastfeeding) SSB and fructose consumption (predominantly fructose) was inversely related to childhood cognitive performance [163,164], social–emotional development [165], and alterations in brain tissue [166]. Prenatal exposure to sucrose in mice was associated with impairments in attention and impulsivity in offspring [167]. Inattention and impulsivity are two of the main characteristics of attention-deficit/hyperactivity disorder (ADHD) [168], stipulated as diagnostic criteria in the DSM-IV [169], and previously linked to diet in human studies [170]. Some age cohort studies found no impact of added sugars on hippocampal brain-derived neurotrophic factor (BDNF) in adult rats where significant alterations were found subsequent to childhood and adolescent SSB intake [50,60]. These results highlight a critical period of increased susceptibility to adverse effects due to sugar consumption, making perinatal, childhood, and adolescence a period of particular vulnerability.

Further research in human models has provided substantial evidence that chronic SSB consumption leads to cognitive impairments over time [8,171] or even after short-term consumption in childhood [44]. A recent systematic review investigating impacts of SSBs on middle-aged and older adults found that only one out of the ten studies identified produced no significant association [171,172]. Negative associations between sugar and cognitive performance were even more profound in diabetic populations [173].

Several researchers have refuted these findings, citing evidence that sugars may in fact have a beneficial impact on cognition [174,175,176,177,178]. These studies’ cognitive tests were administered after fasting of up to 12 h [175,177,178,179,180,181,182,183]. The act of eating breakfast after any period of fasting, independent of the meal constituents, should improve cognitive performance [184,185]. Sugar consumption likely improves performance by replenishing diminishing glucose stores and supporting blood-glucose homeostasis, which diminishes with age as beta cell function deteriorates [186]. This may explain why significant findings are often only evident in the elderly or those with Alzheimer’s disease and memory complaints [178,181,187,188,189]. Evidence in younger adults and children is inconsistent and rarely shows the same improvements in memory or recognition [190,191].

These studies were all conducted within a short timeframe after glucose administration (from 15 min to one hour). Researchers commonly used saccharin as a control substance. Tasks of high cognitive demand have been observed to be facilitated by glucose administration as they utilise more energy than low-demand tasks [180,192,193]. Saccharin and other sugar alternatives cannot be metabolised by the body and would consequently provide no energy for cognitive effort. It would, therefore, be surprising if cognitive performance was equal between energy versus no energy diets among fasted participants. Studies that provided fasted participants a standardised breakfast before sugar or control solutions saw no change, or a reduction, in performance after sugar consumption [176,191,194]. Prior long-term sugar intake of participants, and the impact this may have on results, was rarely considered. The impacts of simple carbohydrates on gut permeability, BDNF levels, or upregulated inflammatory pathways (considered major mechanisms for sugar-induced impairments) were not investigated, nor would short-term consumption necessarily have any significant effect on these systems, which are hypothesised to occur after a longer duration of excessive sugar intake [10,53].

Many reviews that conclude a lack of evidence for the detrimental effects of sugar have failed to consider the studies conducted that have found evidence of significant correlations or profound structural and functional impairments, often relying on evidence that is over 20 years old [12,13,174]. This may be due to a dearth of RCTs in humans relating to this research topic. A recent systematic review [174] that concluded no evidence for a detrimental effect of sugar on cognition described an included study as showing improved recognition speed, spatial and numerical memory, and episodic memory due to glucose administration [193]. However, that study predominantly investigated the role of glucose regulation and found that decreased performance on cognitive tasks was related to poor glucose control, elevated blood glucose, and higher intake of sugar and high-calorie foods and sweets. This would indicate that long term consumption has a detrimental effect on cognition irrespective of short-term impacts. Several studies have supported this finding, revealing a strong effect of glucose control and glucose recovery on cognitive outcomes, highlighting the significant impact of glucose administration response on cognitive function [192,195]. Although these short-term studies are often used to refute claims of the detrimental impacts of sugar on cognitive performance, one could argue that these studies are not testing the same hypothesis. The immediate impacts of glucose on cognitive performance may highlight potential benefits of temporary supplementation but cannot be used to extrapolate long-term impacts.

Observational and experimental studies into the impacts of sugar consumption on cognition are vulnerable to the same limitations of food frequency questionnaires and recall bias as described above. Research should also account for alternative macronutrient intake as high-fat diets have been associated with cognitive impairments and dyslipidaemia [196,197,198], and salt is implicated in high blood pressure [199]. Fibre may have a protective effect due to its role in the microbiome [200,201]. While these factors should be considered in future research, the current evidence strongly indicates a major role for refined sugars in cognitive dysfunction and dementia.

**Table 3:** Findings from published reports on the effects of free or added sugar consumption on cognition in healthy subjects.

Author and Year	Design	Timeframe/ Follow-Up	Subjects	Tasks/Measures	Intervention/ Independent Variable	Significant Findings
Human studies						
Adan & Serra-Grabulosa, 2010 [179].	RCT	0–30 min (unclear)	Fasted adults, aged 18–25y; n = 72; glucose group, n = 18.	RAVLT Purdue-Pegboard JoLO WCST CalCAP Digit Span of WAIS VAS	75 g glucose	↑ Perdue pegboard assembly ↑ Reaction time -No effect of glucose on learning or memory
Azari, 1991 [191].	Double-blind, Repeated measures trial.	30 min	Aged 19–25; n = 18. Fasted with standardized breakfast.	Word list recall and recognition	30 g or 100 g glucose	-No effect of glucose
Benton & Owens, 1993 [202].	RCT	15 min	Young adults, mean age 21y; n = 153	Word list recall Spatial memory test Wechsler story Blood glucose	50 g glucose	-No effect of glucose solution
Brandt, 2015 [177].	Double-blind, placebo-controlled trial. (Glucose compared to aspartame)	15 min	Fasted young adults; mean age 19.47y; n = 41; BMI = 18.5 to 30 kg/m^2^	Word recall task (recognition, recollection or familiarity).	25 g glucose	↓ Familiarity
Chong et al., 2019 [8].	Cross-sectional survey	NA	Adults aged ≥ 60 years	FFQ (total sugars, free sugars, fructose, glucose, sucrose, maltose, lactose) MMSE	↑ Total and free sugar intake.	↓ MMSE score
Flint & Turek, 2003 [203].	Randomised placebo-controlled trial. (Comparison groups: 10, 100, and 500 mg/kg, or 50 g glucose or saccharin placebo)	2 min	Fasted adults aged 18–50 (n = 67)	TOVA program	100 mg/kg glucose	↓ Attention (impaired impulsivity and disinhibition)
Gagnon et al., 2010 [178].	Double-blinded, placebo-controlled trial. (Glucose compared to saccharin)	15 min	Fasting older adults (aged 60 years and over; n = 44)	STROOP Trail making tests A and B Computerised dual task	50 g glucose	↑ Switching ↑ Inhibition ↑ Trail Making Test A, but not B. ↑ Attention
Gui et al., 2021 [44].	Cross-sectional survey	NA	Children; mean age 8.6 years; n = 6387	FFQ (SSB)	↑ SSB consumption	↓ Executive functions ↑ Risk of executive dysfunction
Hope et al., 2013 [176].	Double-blind placebo-controlled experimental trial.	Immediate	Adults; mean age 25.1y; n = 12. Tested after consumption of standardised breakfast.	Erikson Flanker Task	25 g glucose	↓ Sensorimotor processing speed
Kennedy & Scholey, 2000 [180].	Randomised crossover design. (Glucose compared to saccharin)	20 min	Fasted young adults; aged 19–30; n = 20	Serial threes Serial sevens	25 g glucose solution	↑ Performance on Serial Sevens
Macpherson et al., 2015 [181].	Repeated measures RCT. (Glucose compared to saccharin)	5–30 min (unclear)	Fasting young adults; mean age 20.6y; n = 24; Fasting older adults; mean age 72.5y; n = 24	Memory task Tracking task	25 g glucose solution	Older adults: ↑ Recognition memory ↑ Tracking precision Younger adults: No effects
Martin & Benton, 1999 [194].	RCT. 4 block design: glucose vs placebo; fasted vs breakfast (mean 1049 ± 767 kJ; 42.6 ± 30.3 g carbohydrate).	20 min	Female adults; mean age 22.6y; n = 80	Brown–Petersen task	50 g glucose (fasted condition)	↑ Recall
					50 g glucose (breakfast condition)	-No effect of glucose
Miao et al., 2021 [28].	Prospective cohort study (FHS).	19y (mean)	Adults; n = 1384	FFQ (SSB) Clinical surveillance	↑ SSB	↑ Dementia ↑ AD
Munoz-Garcia et al., 2020 [45].	Prospective cohort study	6y	University graduates; aged over 55y; n = 1069	FFQ (SSB) STICS-m	↑ SSB	↓ Cognition
Owen et al., 2010 [182].	Between-participant, double-blind, placebo-controlled design.	15 min	Fasted young adults; aged 18–30; n = 90	Word presentation Immediate word recall Face presentation Implicit memory task Delayed word recall Delayed word recognition Face recognition	25 g glucose	↓ Word recognition (increased errors)
					60 g glucose	↑ Immediate free recall ↑ Word recognition ↑ Implicit memory
Scholey et al., 2009 [183].	RCT. (Glucose compared to saccharin)	20 min	Fasted young adults (mean age 21.6 years; n = 120	Word recognition Tracking task	25 g glucose solution	↑ tracking performance -No effect on memory
Stollery & Christian, 2016 [175].	Experimental. glucose or saccharin (no sugar).	10 min	Fasting adults; n = 31	Object location binding task	30 g glucose	↑ Object location binding memory ↑ Location memory
Sunram-Lea et al., 2011.	Double-blind, placebo-controlled, balanced, crossover trial. (Glucose compared to saccharin)	15 min	Fasted young adults; n = 30	Immediate word recall Serial threes Serial sevens Corsi block-tapping task Delayed word recall Delayed word recognition	15 g, 25 g, 50 g, or 60 g glucose solution	U-shaped dose-response. -Spatial WM, immediate recall, delayed recall, and recognition memory were all improved at 25 g only.
Ye et al., 2011 [46].	Cross-sectional survey.	NA	Aged 45–75y; n = 1500	FFQ (Sucrose, glucose, fructose, galactose, lactose, maltose, fruit juice, or sugar-sweetened solid foods). MMSE Word list learning Digit span Clock drawing Figure copying STROOP Verbal fluency tests	↑ Total sugars/added sugars/sucrose/glucose/fructose	↓ MMSE -No effect of increased natural fructose, galactose, lactose, maltose, fruit juice, or sugar-sweetened solid foods.
Zhang et al., 2022 [47].	Cross-sectional survey	NA	Aged 13–18y; n = 1427	FFQ (SSB) Questionnaire	↑ SSB	↓ Inhibition ↓ WM ↓ Cognitive flexibility
Animal studies						
Beecher et al., 2021 [10].	Longitudinal experimental study. (Sucrose compared to water)	12 weeks	Adolescent mice; n = 46	Elevated-plus-maze Novelty suppressed feeding Marble burying Open field test Forced swimming test NOR MWM Pathology tests	25% sucrose solution	↓ Episodic and spatial memory ↓ Overall density of dentate gyrus proliferating cells ↑ Locomotor activity
Fierros-Campuzano et al., 2022 [48].	Longitudinal experimental study. (Fructose compared to water)	12 weeks	Adolescent male Wistar rats; aged 5–6 weeks; n = 60	Barnes Maze Pathology tests	10% fructose solution	↓ Spatial memory ↓ Neurogenesis in hippocampus ↑ Inflammatory markers in PFC ↑ GFAP expression in hippocampus and PFC
Hamelin et al., 2022 [49].	Longitudinal experimental study. (Sucrose compared to water or artificial sweetener)	6 weeks	Adult male mice; n = 297	Mouse gambling task Pathology tests	1% sucrose solution (25% daily sugar intake).	↓ DA and DA turnover in PFC ↓ Decision-making ↓ c-Fos expression in prelimbic cortex, nucleus accumbens, and striatum. ↑ Activity in BLA
Hsu et al., 2015 [50].	Longitudinal experimental study. (Sucrose or fructose compared to water)	30 days	Adolescent (n = 38) and adult (n = 38) male Sprague Dawley rats.	Barnes maze test Y-maze	SSB (11% sucrose)	Adolescents: ↓ Spatial learning Adults: -No effect observed
					HFCS (11%)	Adolescents: ↓ spatial learning and memory retention ↑ Hippocampal inflammatory markers Adults: -No effect observed
Kageyama et al., 2022 [60].	Longitudinal experimental study.	40 days	Postnatal, adolescent, and adult Sprague Dawley rats (n = 7–8 per group).	Pathology results	20% HFCS	↓ BDNF expression in childhood and adolescence -No effect in adult rats
Lee et al., 2021 [51].	Longitudinal experimental study. (Comparison of high sucrose to high-fat and control diets)	21 days	Older Sprague Dawley rats; 15 months old; n = 36; high sucrose group, n = 17	T-maze	Sucrose as 70% of carbohydrate kcal	↓ Cognitive learning
Lemos et al., 2016 [52].	Longitudinal experimental study.	9 weeks	Male Wistar rats; 12 weeks old; n = 6–8 rats per group.	Open field test Object displacement NOR Forced Swimming test Western Blot	35% sucrose	↓ Memory performance ↑ Inhibitory Adenosine A_1_ receptor in hippocampus
Messier et al., 2007 [204].	Repeated measures RCT. (Comparison of high-fructose diet to high-fat and control diets)	3 months	7-week-old C57BL/6 mice; n = 38; fructose group n = 8	Operant bar pressing task	15% fructose	↑ Learning (on 2 of 5 testing days)
Miles et al., 2021 [205].	Longitudinal experimental study.	14 days	Adult male Wistar rats; 8 weeks old; n = 16	Location Discrimination task Pairwise Discrimination acquisition and reversal learning Processing speed	10% sucrose (approx. 70 mL per day)	-No effect of sucrose
Noble et al., 2019 [53].	Longitudinal experimental study.	30 days (Postnatal day 26 to 56)	Juvenile, male Sprague Dawley rats (n = 24).	Zero Maze Novel object in context task	11% *w/v* HFCS	↓ later-life hippocampal-dependent episodic contextual memory -No impact on glucose tolerance, weight, anxiety
Reichelt et aal., 2022 [57].	Longitudinal experimental study.	28 days	male albino Sprague Dawley rats; 4 weeks old; n = 32	Object-in-place task Locomotor behaviour Biconditional discrimination Immunohistochemistry	200 mL 10% sucrose, 2 h per day.	↓ Context-appropriate responses ↓ Hippocampal PV+ cells
Ross et al., 2009 [206].	Longitudinal experimental study.	18 weeks	Male Sprague Dawley rats; n = 29.	Spatial Water Maze	60% fructose	↓ Retention performance -No impact on acquisitional performance
Sanguesa et al., 2018 [61].	Longitudinal experimental study. (Comparison of fructose, glucose, water)	28 weeks	Female, adult, Sprague Dawley rats; n = 36; control, n = 12; Fructose, n = 12; glucose, n = 12	NOR MWM Immunohistochemistry	10% *w/v* fructose	↓ NOR ↓ BDNF ↓ IRS-2 protein expression ↓ Akt phosphorylation
Wong et al., 2017 [54].	Longitudinal experimental study.	24 days	Adolescent and young adult Sprague Dawley rats; n = 48	Object and place recognition memory Delay-discounting task Progressive ratio T-maze forced alternation.	10% sucrose solution, 2 h per day.	↓ Spatial memory
Wu et al., 2015 [55].	Longitudinal experimental study.	8 months	Male Sprague Dawley rats; 8 weeks old; n = 19	MWM	10% fructose solution	↓ Spatial learning and memory
Xu & Reichelt, 2018 [56].	Longitudinal experimental study.	28 days	Male Sprague Dawley rats; 3 weeks old; n = 36	Open field test NPR NOR Immunohistochemistry	10% sucrose, 2 h per day	↓ NPR ↓ NOR ↓ Hippocampal PV+ cells

Abbreviations: RCT, randomised control trial; mins, minutes; y, years; RAVLT, Rey Auditory Verbal Learning Memory Test; JoLO, Benton Judgement of Line Orientation Test; WCST, Wisconsin Card Sorting Test; CalCAP,, California Computerized Assessment Package; WAIS, Wechsler Adult Intelligence Scale; VAS, Visual Analogue Scale; ↑ increased/increasing; ↓ decreased/decreasing; MMSE, the Mini Mental State Examination; TOVA, Test Of Variables of Attention program; SSB, sugar-sweetened beverage; FHS, Framingham Heart Study; STICS-m, Spanish version of the modified Telephone Interview of Cognitive Status; WM, working memory; NOR, novel object recognition; MWM, Morris Water Maze; PFC, prefrontal cortex; GFAP, Glial fibrillary acidic protein; DA, dopamine; BLA, basolateral amygdala; HFCS, high-fructose corn syrup; AD, Alzheimer’s disease; BDNF, brain-derived neurotrophic factor.

### 2.7. Mood

The impact of sugar on mood and behaviour is less certain and findings are frequently inconsistent, although some hypothesise that any adverse impacts may be due to the neuropathological effects of sugars [207]. As with the cognition studies described above, many previous studies on mood have investigated only the short-term impacts of sugar (generally 30–60-min following glucose administration) and also after a prolonged period of fasting [187,208,209,210,211]. Hypoglycaemia has been observed to have an adverse effect on both mood and cognition and would provide a poor control group while being potentially reversed by administration of any carbohydrate [212,213].

Where retrospective and longitudinal methods have been employed, significant correlations have been found between diets high in sugar and major depression or depressive symptoms [214,215]. A large Chinese study found that prevalence of depressive symptoms was doubled in those consuming four cups or more of SSBs (specifically soft drinks or soda) per week compared to those who consumed less than one cup [9]. Another study found a 60% greater risk in depression and suicidal ideation in those consuming more than 500 mL of SSBs per day [216]. Other studies indicate a role for high-sugar diets in anxiety, stress, hyperactivity, and conduct issues [10,158,207,217]. However, the impacts of sugars on psychological health are still unclear, and studies must contend with the large number of confounders, subjectivity, and potential malingering that complicate mental health research. Further research is needed to clarify the relationship between added sugars and psychological health.

## 3. Possible Mechanisms of Action

It is hypothesised that impacts of chronic high sugar consumption on mood are a subsequent effect of the neurological impacts of sugar consumption. Western diets have been associated with systemic inflammation, neuroinflammation, and reduced BDNF in the hippocampus, impairing synaptic plasticity, executive function, and mental health [214,218]. Sugar has also been found to induce neurochemical changes in the central nervous system, specifically changes to dopamine signalling and sensitization of D-1 dopamine and mu-1 opioid receptors. Subsequent removal of high-fat and high-sugar foods leads to anxious and depressive behaviours due to reductions in dopamine levels in the NAcc [219,220]. These same withdrawal symptoms and neurochemical changes are observed in drugs of dependence [58,219,221].

### 3.1. Addiction and Dopaminergic Alterations

Mechanisms and signals involved in metabolic and homeostatic control can be disrupted via several proposed mechanisms. Consumption of palatable foods, including sugar, stimulates dopamine release in the ventral tegmental area (VTA) of the hypothalamus, activating reward pathways (from the VTA to the NAcc), which can override satiety signals [165,166]. Repeated exposure to palatable foods alters mesocorticolimbic dopamine circuitry, dysregulating homeostatic controls, reinforcing food cues, and increasing feeding [167,168]. These dopaminergic signalling pathways are thought to be crucial for reward motivation and memory, specifically episodic and working memory [169]. Activation of reward pathways leads to increased sugar seeking and consumption. Alterations in dopamine signalling can lead to reduced plasticity in the NAcc, which is hypothesised to cause the memory impairments observed in cases of addiction [222].

In rat models, excessive sugar consumption elicits signs of addiction, with bingeing, withdrawal, depressive-like behaviours, increased reward seeking, and higher resilience to foot shock punishments than methamphetamines [10,223]. Intermittent sugar consumption produced an increase in extracellular dopamine in the NAcc and decreased enkephalin mRNA expression and opioid modifications associated with withdrawal [219]. Sugar also activates the hypothalamus (the principal regulator of satiety and hunger behaviours) and inhibits ghrelin and leptin production, reducing the sensation of being full and promoting overconsumption [10,224].

### 3.2. Microbiome Disruption and Neuroinflammation

Several disease states are thought to be caused by disruption of the gut microbiome (dysbiosis) brought about by poor diet. Dysbiosis has been implicated in pathogenesis of obesity, insulin resistance, and non-alcoholic fatty liver disease [225]. High-fat and high-sugar diets impair gut wall permeability by reducing numbers of protective microbiota, impacting intestinal mucosa, disrupting tight junctions, and increasing bacterial translocation, leading to increased inflammatory cytokine signalling [24,226]. Sugar alone has been associated with profound dysbiosis of gut microbiome, and alterations are similar to those observed in neurodegeneration [227,228,229]. Human and animal studies have shown significant alterations in gut microbiome due to sugar consumption. One study by Jones et al. [230] found that dietary fructose, and no other dietary macronutrients, impacted microbiota, with a significant inverse association between fructose and Eubacterium eligens and Streptococcus thermophilus, two beneficial microbes considered to have anti-inflammatory properties and promote gut health. Rodent studies using high-fructose diets have observed dramatic shifts in microbial colonies, increases in pro-inflammatory cytokines, reductions in anti-inflammatory cytokines, lipid accumulation in the liver, and neuroinflammation [24,228]. These impairments were observed to occur independent of body weight or caloric intake [53].

Structural impairment to gut wall permeability enables entry of liposaccharides into the blood stream, activating Toll-like receptor-4 and leading to excessive production of proinflammatory cytokines [231,232]. This low-grade systemic inflammation is known as metabolic endotoxemia and can lead to several chronic inflammatory conditions [229]. Fructose-induced dysbiosis-triggered hippocampal neuroinflammation and neuronal loss in mice may highlight a potential mechanism for the neurological and psychiatric impairments associated with sugar and obesity [224,228,233].

## 4. Strengths and Limitations

This paper has several strengths. While it is not a systematic review, a thorough search of PubMed, Embase, and Google Scholar was conducted, and every attempt was made to search for all systematic reviews and meta-analyses that exist on the topics discussed. This paper covers several aspects of health that may be impacted by sugar intake and thoroughly examines and critiques many substantial arguments around dietary factors in health and wellbeing. Several health conditions that may also be impacted by sugar, such as cancer, kidney disease, and dental caries, were not included as the authors chose to focus on the most commonly contested issues in order to deliver a more focused and comprehensive discussion. We aimed to provide an up-to-date and in-depth examination of the literature; investigating the nuances of macronutrient intake, approaches to measurement, data collection, and methodologies that have impacted previous findings and current understandings of the research.

A major limitation of the studies discussed, when describing human dietary studies, is the requirement to rely on regular participant recording (food diaries) or recall of food intake (in dietary questionnaires or interviews), as well as assuming a certain consistency in portion size interpretation between participants or groups. It is common for participants to greatly underestimate alcohol intake, which is a large contributor to overall sugar and calorie consumption [234].

Use of the terms carbohydrates and sugars is often disparate between study types. As new research elucidates the importance of glycaemic index and the potential differences in physiological influence of different sugar types on neurological and biological processes, the importance of classifying and controlling for diet types becomes particularly important. The terms added or free sugars are not considered to include those from dairy, vegetables, or whole grains. However, these are sometimes referred to or included in total sugar estimates, which can provide highly misleading conclusions. For example, Sievenpiper describes an observed negative correlation between diabetes incidence and consumption of high-fibre cereals, grains, and nuts as a protective effect of sucrose consumption [11,140]. Many studies fail to collect or disclose carbohydrate class or source (i.e., carbohydrates from table sugar, oats, fruit, or Coca Cola could all lead to vastly different determinations), making interpretation and comparative evaluation problematic.

## 5. Conclusions

In conclusion, very little scientific evidence exists that indicates a benefit of added dietary sugars; however, an overwhelming and growing body of evidence highlights the negative effects of excessive or prolonged sugar intake. This is particularly significant for fructose and high-fructose corn syrup. There may be benefits to glucose supplementation for some individuals in times of increased cognitive requirements. However, glucose can be acquired from healthy dietary sources, such as fruit, vegetables, and whole grains, which all provide nutritional benefits to the body. There is also little evidence that all added sugars must be eradicated from the diet. However, the current guidelines of limiting energy intake consumed as added sugars to 5–10% have so far held up under scrutiny, particularly when considering the high disease burden and significant financial impacts of sugar-induced dental disease [235].

The above review has highlighted the significant differences between carbohydrate classes and their potentially diverse impacts on health and different population groups. The independent impacts of macronutrients, and the artificial sweeteners that have frequently replaced them, need to be further explored. There is also the potential for sugar-associated impairments, particularly regarding impacts on cognition and developmental conditions, to impose a greater challenge for those with an underlying predisposition (e.g., a mental health condition) or an existing condition (such as ADHD). Large-scale population studies are not the ideal methodology to identify individualised impacts of different macronutrients and macronutrient diet combinations on various population groups. RCTs and cohort studies in a diverse range of participants, measuring precise macronutrient composition and mediating factors, such as exercise, must be conducted.

## Figures and Tables

**Table 1:** Summary of findings outlining the effects of added sugar consumption.

Condition	Relationship	Impact on Related Systems
CHD [14,15,16,17,18]	↑	Dyslipidaemia [15,19,20,21,22,23] Increased pro-inflammatory cytokines [15,23,24] Reduced insulin sensitivity [23]
Stroke [25,26,27,28]	↑	
T2DM [29,30,31,32,33,34,35,36,37,38]	↑	Dyslipidaemia [15,19,20,21,22,23] Hyperglycaemia [21] Increased adipose tissue [21,39] Increased de novo lipogenesis [21] Increased liver fat [39,40] Reduced insulin sensitivity [23]
NAFLD [40]	↑	
Metabolic Syndrome [41,42,43]	↑	
Executive function [8,10,28,44,45,46,47,48,49,50,51,52,53,54,55,56]	↓	Hippocampal dysfunction [10,48,50,52,57,58] Microbiome dysbiosis [24,59] Neuroinflammation [48,50] Reduced Brain-Derived Neurotrophic Factor (BDNF) expression [60,61] Alterations in dopaminergic signalling [49,62]
Obesity [21,37,43,63]	↑	Increased adipose tissue [21,39] Reduced insulin sensitivity [21,22] Alterations in dopamine signalling [49,62]

Abbreviations: CHD, coronary heart disease; T2DM, type 2 diabetes mellitus; NAFLD, non-alcoholic fatty liver disease; ↑ increased/increasing; ↓ decreased/decreasing.

## Data Availability

Not applicable.
